# inDAGO: a user-friendly interface for seamless dual and bulk RNA-Seq analysis

**DOI:** 10.3389/fbinf.2025.1696823

**Published:** 2025-11-21

**Authors:** Gaetano Aufiero, Carmine Fruggiero, Nunzio D’Agostino

**Affiliations:** Department of Agricultural Sciences, University of Naples Federico II, Portici, Italy

**Keywords:** transcriptomic dynamics, gene expression profiling, differentially expressed genes, graphical user interface (GUI), R-shiny framework, cross-species RNA-seq

## Abstract

Dual RNA-sequencing enables simultaneous profiling of protein-coding and non-coding transcripts from two interacting organisms, an essential capability when physical separation is difficult, such as in host-parasite or cross-kingdom interactions (e.g., plant-plant or host-pathogen systems). By allowing *in silico* separation of mixed reads, dual RNA-seq reveals the transcriptomic dynamics of both partners during interaction. However, existing analysis workflows often require programming expertise, limiting accessibility. We present inDAGO, a free, open-source, cross-platform graphical user interface designed for biologists without coding skills. inDAGO supports both bulk and dual RNA sequencing, with dual RNA sequencing further accommodating both sequential and combined approaches. The interface guides users through key analysis steps, including quality control, read alignment, read summarization, exploratory data analysis, and identification of differentially expressed genes, while generating intermediate outputs and publication-ready plots. Optimized for speed and efficiency, inDAGO performs complete analyses on a standard laptop (16 GB RAM) without requiring high-performance computing. We validated inDAGO using diverse real datasets to demonstrate its reliability and usability. inDAGO, available on CRAN (https://cran.r-project.org/web/packages/inDAGO/) and GitHub (https://github.com/inDAGOverse/inDAGO), lowers the technical barrier to dual RNA-seq by enabling robust, reproducible analyses, even for users without coding experience.

## Highlights

- inDAGO is a user-friendly, cross-platform software tool that enables both dual and bulk RNA-seq analysis through a graphical user interface (GUI), removing the need for programming skills and lowering the barrier for non-bioinformaticians.
- Supports both sequential and combined approaches for dual RNA-seq, offering flexibility in genome indexing, read mapping, and read discrimination, making it suitable for diverse interspecies interaction studies.
- Implements a complete RNA-seq workflow, from quality control and filtering to mapping, summarization, exploratory data analysis, and differential gene expression (DEG) identification, optimized for standard laptops with at least 16 GB RAM.
- Promotes the democratization of bioinformatics by offering an open-source, R-Shiny-based solution that enables researchers without coding experience to perform complex transcriptomic analyses.

## 1 Introduction

When studying tissues composed of material from two distinct organisms, such as host–pathogen, host–symbiont, or plant–parasitic plant interfaces, transcriptome profiling typically follows one of two main strategies: (i) physical separation of the constituent tissues or (ii) direct sequencing of the mixed sample followed by computational assignment of reads. Physical separation methods, including laser capture microdissection (LCM) or manual microdissection followed by RNA extraction and sequencing, minimize cross-contamination between organisms and allow unambiguous transcript assignment. However, these approaches require specialized instrumentation and trained personnel, and they can be technically challenging or impractical when the interface is structurally complex (Honaas et al., 2013; Jhu and Sinha, 2022).

Alternatively, RNA can be sequenced directly from the mixed tissue, with sequencing reads computationally assigned to each organism by mapping to their respective reference genomes or transcriptomes. This strategy, commonly referred to as dual RNA-sequencing (RNA-seq), avoids laborious microdissection and enables simultaneous profiling of all interacting partners (Westermann et al., 2012). However, it introduces analysis-specific challenges, including ambiguously mapped reads, extreme abundance imbalances that reduce sensitivity for low-abundance transcripts, and dependence on the completeness and quality of reference sequences for accurate organismal assignment (Fruggiero et al., 2024).

To date, the dual RNA-seq approach has been employed to study a broad spectrum of interspecies interactions, ranging from mutualism (Mateus et al., 2019) to host-pathogen dynamics (Westermann et al., 2017; Naidoo et al., 2018) and covering nearly all taxonomic kingdoms, including bacteria, fungi, plants and animals. Dual RNA-seq can be conducted following two main approaches: combined and sequential. Although both approaches share the fundamental steps of traditional bulk RNA-sequencing (bulk RNA-seq) analysis (i.e., read quality control and preprocessing, reference genome indexing, read mapping, data summarization, exploratory data analysis, and identification of differentially expressed genes (DEGs)), they differ in several critical aspects. In the sequential approach, the genomes of the interacting species are indexed separately, while in the combined approach, all genomes are concatenated and indexed as a single entity. The combined approach produces a unified sequence alignment file (SAM/BAM/CRAM format) after mapping, whereas the sequential approach generates separate alignment files for each species. Therefore, the alignment results from the combined approach needs to be carefully partitioned to extract species-specific data (Espindula et al., 2019; Fruggiero et al., 2024). These studies also compared sequential and combined read-mapping strategies, finding that the combined approach generally provides modestly improved performance. In summary, dual RNA-seq shares many similarities with traditional bulk RNA-seq, with the key differences primarily occurring in the genome indexing process and the handling of alignment files before summarization. Bulk RNA-seq analysis can be performed via the command line by combining various software tools, enabling users to design custom pipelines in different programming languages. Additionally, several graphical user interfaces (GUIs) have been developed to streamline this process, including platforms such as RNASeqGUI (Russo and Angelini, 2014), Galaxy (Afgan et al., 2018), RNAdetector (La Ferlita et al., 2021), and RNAlysis (Teichman et al., 2023). Despite the wide range of tools available for bulk RNA-seq analysis, no dedicated platforms have been developed specifically for dual RNA-seq analysis. Currently, performing dual RNA-seq analysis requires the development of customized pipelines, which demand proficiency in programming and scripting. This presents a challenge for scientists who lack programming skills but wish to use a user-friendly interface that guides them through the analysis process step by step. To address this gap, this article introduces inDAGO, a standalone software with a GUI designed to automate dual RNA-seq analysis and assist users at every stage of the process. Unlike other RNA-seq GUIs, inDAGO natively implements dual indexing and mapping strategies, transparently handles cross-mapped and unmapped reads, and integrates exploratory modules that allow users to examine species-specific signals before performing differential expression analyses. The software packages the entire workflow into a user-friendly interface that ex-ports standard, publication-ready outputs. inDAGO is compatible with multiple operating systems and can run on either a high-performance server or a laptop with at least 16 GB of RAM. No programming expertise is needed, allowing users to smoothly navigate the steps of the analysis and download intermediate results as required. Furthermore, inDAGO includes additional modules for performing classical bulk RNA-seq analysis, making it a versatile tool for a wide range of RNA-seq applications.

## 2 Materials and methods

### 2.1 Functionality overview

**FIGURE 1:**
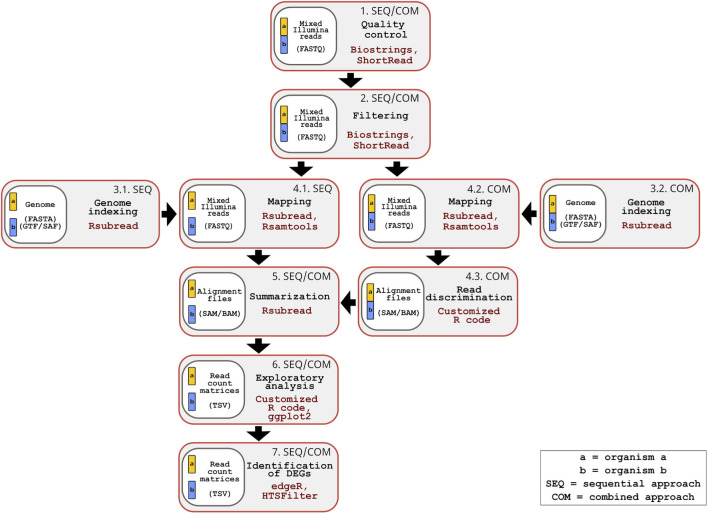
Overview of the inDAGO dual RNA-seq workflow supporting sequential and combined mapping approaches. The workflow consists of seven steps, with steps 1, 2, 5, 6 and 7 being common to both approaches, while steps 3 and 4 are approach specific. Step 1: Quality control of raw mixed reads (organism a+ organism b in FASTQ format) is performed using the Biostrings and ShortRead packages and graphical results are generated through ggplot2 and custom R scripts. Step 2: Filtering of raw mixed reads is performed using the Biostrings and ShorRead packages (Input format: FASTQ). Step 3: Genome indexing of reference sequences (Input format: FASTA and GTF or SAF) is performed with the Rsubread package and built-in R functions. In the sequential approach, separate genome indexing is done for each organism (Step 3.1), while in the combined approach, a single concatenated genome is indexed (Step 3.2). Step 4: Alignment of filtered reads to the reference genomes, their manipulation and the *in silico* discrimination of mixed transcripts are conducted using Rsubread, Rsamtools and built-in R functions. In the sequential approach, a double mapping step is performed (one for each organism) (Step 4.1), while in the combined approach, a single mapping is followed by *in silico* read discrimination (Steps 4.2 and 4.3) (Input format: FASTQ and SAM/BAM). Step 5: Mapped reads are summarized using the Rsubread package (Input format: SAM/BAM). Step 6: Summarized reads are explored through statistical analysis and visualizations using custom R code, along with the ggplot2, pheatmap, Hmisc, and RNAseQC packages (Input format: TSV; TAB-separated values). Step 7: Differentially expressed genes (DEGs) are identified using the edgeR and HTSFilter packages (Input format: TSV). The genomes of the two organisms are represented in different colors (yellow and blue). When analyzed separately, each genome appears as an independent block in the workflow. When analyzed together, the genomes are displayed as connected blocks.

**FIGURE 2:**
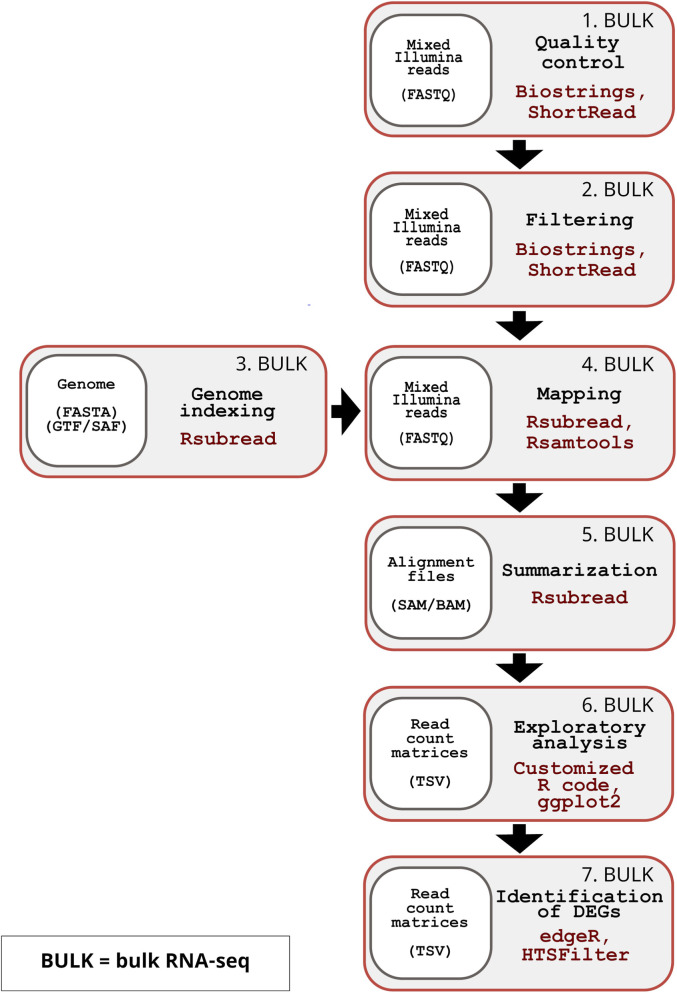
Overview of the inDAGO bulk RNA-seq workflow. The inDAGO workflow for bulk RNA-seq analysis consists of seven key steps that trace the full analytical process, ultimately leading to the identification of differentially expressed genes (DEGs) between experimental conditions. Step 1: Quality control of raw reads (Input format: FASTQ). Step 2: Filtering of low-quality reads (Input format: FASTQ). Step 3: Indexing of reference genome (Input format: FASTA and GTF or SAF). Step 4: Alignment of reads to the reference (Input format: FASTQ). Step 5: Summarization of mapped reads by biological units (e.g., genes) (Input format: SAM/BAM). Step 6: Statistical exploration of read counts (Input format: TSV). Step 7: Identification of differentially expressed genes (Input format: TSV). The bulk RNA-seq workflow follows the same sequence and uses the same underlying packages as the dual RNA-seq workflow.

**FIGURE 3:**
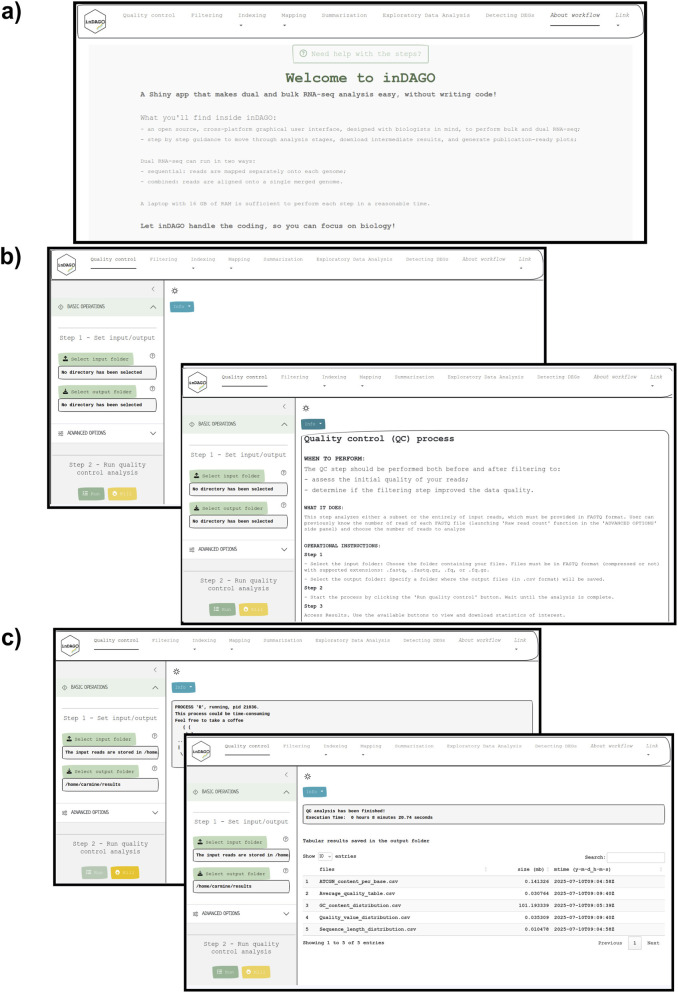
Graphical User Interface of inDAGO. Screen captures from inDAGO illustrating key stages of the analysis workflow: **(a)** welcome screen; **(b)** quality control opening screen, with the built-in documentation screen overlapping; **(c)** module view during process execution, with the display of results after analysis completion overlapping. Together, these images provide a comprehensive overview of inDAGO’s user-friendly interface and functionality.

The inDAGO software is fully implemented in the R programming language (R Core Team, 2024), with its GUI developed using the Shiny application framework (Chang et al., 2025). It is compatible with GNU-Linux, Windows, and macOS operating systems. As illustrated in Figure 1, inDAGO offers a seamlessly integrated analysis workflow that supports both sequential and combined approaches for key steps, including indexing, mapping, and read discrimination (the latter applied only in the combined approach). This flexibility enables the workflow to be tailored to the specific needs of dual RNA-seq experiments. Another key strength of inDAGO is its dedicated modules for bulk RNA-seq analysis, covering the entire workflow from quality control to the final stages of analysis (Figure 2). In addition to supporting bulk RNA-seq, inDAGO offers specialized tools for managing unmapped reads (sequences that do not align to any reference genome). These unmapped reads can be further analyzed by mapping them to alternative reference sequences, aiding in the detection of potential contaminants, such as reads originating from unintended organisms. Additionally, inDAGO enables remapping of previously unmapped reads by adjusting mapping stringency parameters, such as the allowed number of mismatches or the treatment of multi-mapped reads, thereby improving read assignment accuracy. This flexibility enhances inDAGO’s utility for both dual and bulk RNA-seq workflows. inDAGO consists of seven distinct modules: (1) quality control, (2) filtering, (3) genome indexing, (4) mapping, (5) summarization, (6) exploratory data analysis (EDA), and (7) identification of DEGs. The layout and functionality of the GUI is shown in Figure 3, reporting the quality control module for illustrative purposes. Navigation between modules is streamlined via tab panels and drop-down menus, allowing users to quickly access any desired analysis step. To run the inDAGO pipeline starting from the quality assessment module, paired-end reads in FASTQ format are required, along with the reference genome and annotation files (FASTA and GTF or SAF) for one or both species, depending on whether a bulk or dual RNA-seq analysis is performed. Each module automatically generates additional output files, including BAM alignments and results from DEG analysis. The workflow is specifically optimized for paired-end reads, as single-end reads were excluded due to their lower mapping accuracy and reduced reliability in dual-origin or complex transcriptomes. Additionally, ongoing analysis can be terminated and restarted at any time, offering greater flexibility and control over the workflow. The inDAGO package is freely available through both CRAN (https://cran.r-project.org/web/packages/inDAGO/index.html) and the GitHub repository (https://github.com/inDAGOverse/inDAGO).

### 2.2 Methodology

#### 2.2.1 Quality control

The module is designed to process paired-end reads obtained from the Illumina sequencing platforms in FASTQ format. It extracts essential information from these files and should be run both before and after the filtering step to assess the reliability of the reads and evaluate whether filtering has improved the data quality. The quality control process mainly uses functions from the ShortRead (Morgan et al., 2009) and Biostrings (Pagès et al., 2025) packages to efficiently handle FASTQ files and process quality scores, and ggplot2 (Wickham, 2009) and custom R code to produce the graphical outputs. The module enables analysis of all reads, though this can be computationally intensive, or allows examination of a random subset of records from each input FASTQ file. It also provides a preliminary count of reads in each input file. To accelerate processing, users can specify the number of cores for parallel computation. Results are generated in tabular format, with additional options to visualize and download graphical representations of the data in three formats, displaying either all samples or a selected subset. The module offers a comprehensive overview of key quality metrics, including base quality and distribution, read length distribution, GC content, and base composition. These metrics are visualized through line plots, boxplots, area charts, and density plots, providing a clear assessment of sequencing data quality and characteristics. This information supports informed decisions for downstream analyses.

#### 2.2.2 Filtering

The filtering module processes raw sequencing data stored in paired-end FASTQ format, improving read quality through multiple refinement steps. It enhances data quality by (1) trimming low-quality bases from the ends of each read; (2) applying a sliding window approach to filter out sequences with a high proportion of low-quality bases; (3) removing adapter sequences. The filtering process, as well as quality control, also uses functions from the ShortRead and Biostrings packages. To ensure accurate read retention, the module implements several key parameters. First, bases below the defined quality threshold are identified and excised from both ends until only high-quality bases remain. A sliding window analysis is performed along each read, examining a set number of neighboring bases; if too many low-quality bases appear in sequence, trimming occurs at that point. Then, known adapter sequences are detected and clipped out with allowance for a small, controlled number of mismatches, ensuring that contamination is removed without sacrificing genuine sequence data. Finally, sequences shorter than the specified minimum read length are removed. The module allows adjustment of quality score formats to ensure compatibility with different sequencing platforms, either via automatic detection or by selecting a predefined format. Performance and storage efficiency can be optimized by enabling file compression, which generates ‘.gz’ files to reduce disk usage. Processing speed can be improved by parallelizing the analysis, distributing tasks across multiple jobs for faster execution. Additionally, datasets can be divided into smaller sub-samples for sequential processing, helping to balance computational resources, memory usage, and filtering efficiency. After processing, the module outputs the filtered reads in FASTQ format, ensuring high-quality data for downstream analysis.

#### 2.2.3 Genome indexing

The genome indexing module begins with reference genomes in FASTA format (which can be provided in compressed or uncompressed forms using the GNU-Linux *gzip* utility) and constructs a data structure that enables efficient access to the genome.

A hash-table data structure is generated from the reference genome file, enabling rapid and efficient read alignment while significantly reducing processing time. The indexing processes utilize the *buildindex* functions from the Rsubread (Liao et al., 2019) package. This module comprises three sub-modules, supporting dual RNA-seq analysis in both sequential and combined modes, as well as conventional bulk RNA-seq analysis. In sequential mode, the module generates separate genome indexes for each target organism, ensuring independent alignment. In contrast, the combined mode creates a single index by concatenating the genomes of the interacting organisms, assuring a unified mapping process. The required inputs include the two genomes for dual RNA-seq or a single genome for bulk RNA-seq, which must be indexed before the mapping step. In the combined dual RNA-seq approach, inDAGO allows us to assign distinct prefixes to each genome to ensure clear labelling of genome sequences. The module allows configuration of advanced parameters for filtering repetitive sequences and splitting the index into chunks when available RAM is insufficient for a single block. Additionally, indexing can be performed on one genome at a time or on both genomes simultaneously. This option is available only in sequential mode, when generating two separate indexes is required. The output consists of two binary index files for each index block, which are saved in the designated folders, along with three plain text documents.

#### 2.2.4 Mapping

Similarly to the indexing module, the mapping process varies depending on the chosen analysis approach. In all cases, reads are aligned to the specified reference sequences, generating alignment files in SAM/BAM format, while unmapped reads are output in FASTQ format. The mapping processes utilize the *subjunc* functions from the Rsubread to perform read alignment. While the manipulation of resulting BAM files is performed using *Rsamtools* (Morgan et al., 2025) package and the generation of the concatenated genome and the *in silico* separation of mixed reads is conducted using R built-in functions.

For the combined approach, aligned reads are assigned to their respective reference sequences, and cross-mapped reads (those that align to multiple genomes) are also output in BAM format.

The sequential approach requires two separate genome indexes, whereas the combined approach and bulk RNA-seq require only a single index. These indexes, along with input reads in FASTQ format, serve as the necessary input for the mapping process. The analysis can be fine-tuned by adjusting parameters that affect mapping stringency, handling of multi-mapped reads, and processing speed. Alignment outputs can be generated in either uncompressed or compressed formats, with the number of cores and threads configurable for parallel processing. The results consist of two BAM files: one for each reference sequence in the dual RNA-seq approach, and a single BAM file for bulk RNA-seq. In the combined approach, a single mapping is performed, resulting in a preliminary alignment file. This file is then processed and split according to each interacting organism, generating two organism-specific BAM files. Additionally, cross-mapped reads are stored in a separate output BAM file. Unmapped reads are provided in FASTQ format. Furthermore, three text files are generated that detail the discovered junctions, indels, and a summary of the analysis.

#### 2.2.5 Summarization

The summarization process uses the *featureCounts* function from the Rsubread package to assign mapped reads (i.e., SAM/BAM files) to specific genomic features and quantifying gene expression for each sample. It generates a count table that reports the number of reads mapped to each selected feature (e.g., exon). Starting from this step onwards in dual RNA-seq, the organisms can be analyzed independently, as the previous module has already differentiated the reads between the two organisms. To operate, the module requires alignment files and an annotation file in GTF/SAF format. Multiple customizable parameters allow users to adjust how annotation data is processed, providing flexibility to tailor the analysis to specific experimental needs. The module retrieves the chosen genomic feature (e.g., exons) and its identifier (e.g., gene_id) from the appropriate annotation file columns. Read counts can be aggregated either across entire genes or at the level of individual features, such as exons. Further customizable parameters offer control over read assignment, handling of multi-mapped reads, quality filtering, paired-end read management, and parallel processing. Strand-specific read counting can be enabled if a strand-specific sequencing protocol was used, ensuring correct transcript quantification. The module produces four key output files per sample: an annotation file, a quantification file, a summary of the quantification process (in CSV (comma-separated values) or TSV formats), and a log file documenting the workflow. Together, these files offer a comprehensive and structured foundation for downstream analysis.

#### 2.2.6 Exploratory data analysis

The primary objective of EDA is to examine and interpret data in relation to the specific biological question under investigation. This process allows researchers to refine their research questions or determine whether additional data collection is needed when existing data are insufficient. This module generates a series of exploratory plots that are essential for identifying patterns, detecting potential outliers, and visualizing key insights. These visualizations not only enhance data comprehension but also serve as powerful communication tools when presenting findings in scientific publications. The module exploits the ggplot2, pheatmap (Kolde, 2025), and RNAseQC (Dufort, 2025) packages to generate the primary visualizations, while the plotly (Sievert, 2020) package is used to create interactive versions for dynamic visualization. The required inputs include count matrices generated during the summarization step for each sample, as well as a matrix indicating the group assignments for each sample. Alternatively, group assignments can be manually defined via the interface. Group assignment refers to categorizing samples into different groups based on experimental conditions or other relevant factors. This is essential in data analysis, particularly in biological and clinical studies, where comparisons between groups help identify meaningful differences. The module offers full interactive control over plot appearance, including a wide range of color-palette options. It also supports exporting figures in multiple formats (JPEG, PNG, TIFF, EPS, SVG, PDF), customizable dimensions (height and width in inches, cm, mm, or pixels), and adjustable resolution. Plots are generated twice (i.e., before and after normalization) allowing for a visual comparison of the normalization effect, as the module also performs count normalization. To explore and visualize the variability and structure within gene expression data, various techniques are implemented in inDAGO. These include dimensionality reduction methods like Multi-Dimensional Scaling (MDS) and Principal Component Analysis (PCA), which help to reveal patterns across samples or conditions. Moreover, the distribution of count data, such as log-transformed values, is visualized using boxplots, while library sizes are represented using bar plots. Additionally, heatmaps can be used to examine variability in gene expression or correlation between samples. Finally, saturation plots can be generated to evaluate the extent of gene detection.

#### 2.2.7 Identification of DEGs

The final module of the workflow begins by utilizing the same data from the EDA module and concludes by generating a list of differentially expressed genes along with key statistical parameters, including log_2_ fold change, p-value, and adjusted p-value which is output in CSV format.

In addition to DEG list, two plots are provided to visualize estimated dispersion: one based on the negative binomial distribution and the other representing quasi-likelihood dispersion. The process starts with importing the raw count data, followed by constructing a design matrix to represent the experimental design. The design matrix contains rows corresponding to samples and columns representing experimental parameters, with values coded as 0s or 1s (1 indicating that a sample is assigned to a specific condition, and 0 otherwise). Depending on the experimental setup, the design matrix can include an intercept term. Currently, inDAGO uses an intercept-free design matrix. Low-abundance genes are filtered out, as they provide limited statistical power for distinguishing between the null and alternative hypotheses. Next, the data is normalized to account for variations such as sequencing depth, and dispersion parameters calculated. Dispersion is a crucial parameter in RNA-seq data analysis, as it quantifies variability and is essential for identifying DEGs. After data pre-processing, hypothesis testing is conducted using the defined contrasts. These contrasts define the pairwise comparisons between experimental groups, enabling the module to identify DEGs. To run the module, users need a directory containing raw count data, a text file specifying sample group assignments (or alternatively, assignments can be made interactively), and an empty directory for output storage. Input contrasts must also be defined to guide comparisons between conditions. Additionally, advanced parameters are available for further customization of the analysis. These parameters allow fine-tuning of filtering thresholds, normalization methods, and other statistical settings to meet specific research needs. Two filtering methods are available. The first retains genes that meet a minimum read count threshold across samples, as described by (Chen et al., 2016). The second determines a threshold based on the pairwise Jaccard similarity index between replicates within each experimental condition, as outlined by Rau et al. (2013) and implemented in the R package HTSFilter. For statistical testing, the module utilizes edgeR (Chen et al., 2025), supporting three methods: exact tests (exactTests), quasi-likelihood F-tests (QLFTest), and gene-wise likelihood ratio tests (LRT). Several normalization approaches are available, including TMM (trimmed mean of M-values) by Robinson and Oshlack (2010), TMMwsp (a variant optimized for datasets with many zeros), RLE (relative log expression) proposed by Anders and Huber (2010), and the upper quartile method (Bullard et al., 2010). For multiple testing corrections, various p-value adjustment methods are available, including Bonferroni, Holm, Hochberg, Hommel, and FDR (false discovery rate). Thresholds for both absolute log2 fold change (log2 FC) and adjusted p-value can be specified to filter the list of DEGs. Moreover, it is possible to merge all DEGs results in a single merged table, and adding information retrieved from columns of annotation file, such as “seqname”, “attribute”, “description” etc. In addition, the module enables the formulation of customizable volcano plots for each comparison, as well as UpSet plots with the same customization options provided in the EDA module. These visualizations are generated using the ggplot2 and UpSetR (Conway et al., 2017) packages. To make the plots interactive, the plotly and upsetjs (Gratzl, 2022) packages are also utilized.

### 2.3 Graphical structure and dynamic documentation

Each module consists of a sidebar and a main panel, with the sidebar guiding data input and parameter settings. The main panel provides real-time feedback on the process status, displaying notifications such as warnings, errors and execution time. If the module generates graphical outputs, they will be presented here, along with options for interactive modifications and downloads of the plots. Each module provides concise, readily accessible documentation to support the analysis workflow. Within the main panel, the documentation is clearly organized into several sections:

- “WHEN TO PERFORM”: this section outlines the specific circumstances or scenarios, where using the module is appropriate;
- “WHAT IT DOES”: this section provides a brief description of the analysis performed by the module;
- “OPERATIONAL INSTRUCTIONS”: this section provides a step-by-step guide on how to execute the analysis effectively;
- “RESULTS”: describes the expected outcomes and the formats in which they will be presented;
- “ADDITIONAL NOTES”: lists any supplementary options and features available.

Additionally, to improve interaction with the documentation, each sidebar input box features a question mark icon (tooltip) that provides detailed, context-specific information, enhancing both clarity and usability.

### 2.4 Evaluation of inDAGO performance

To evaluate its usability and performance, inDAGO was tested on three different machines, one running the Windows 11 with an Intel® Core™ i5-9300HF processor and 16 GB of RAM (2,400 MHz), the second running the GNU/Linux distribution Ubuntu 20.04.6 LTS (Focal Fossa) with an Intel® Core™ i7-1185G7 processor and 16 GB of RAM (1,600 MHz), and the last running the macOS Sequoia version 15.5 with an Apple M1 chip and 16 GB of RAM (2,666 MHz). Since it is not feasible to compare the entire inDAGO workflow against a fully validated real-world project, we divided the workflow into three distinct blocks for evaluation. The first block focused on quality control, filtering, indexing, and mapping using the combined approach. The second block evaluated the summarization process, while the final block assessed data exploration and the identification of differentially expressed genes.

#### 2.4.1 Dataset 1 - Dual RNA-seq mapping validation (*Arabidopsis* - *Cuscuta*)

To evaluate the quality control, filtering, indexing and mapping modules (combined approach mode), we reproduced the dual RNA-seq mapping experiment described by Fruggiero et al. (2024) which simulated the interaction between *A. thaliana* (host plant) and *C. campestris* (parasitic plant). Further methodological details and complementary analyses can be found in the referenced publication. RNA-seq data were retrieved from the European Nucleotide Archive (ENA) and include *A. thaliana* stem tissue samples (accession numbers: SRR22559142, SRR22559143, SRR22559144) from the Columbia ecotype (Col-0) at the vegetative stage, with an average of ∼20.1 million reads per sample. Additionally, *C. campestris* developing haustoria samples (accession numbers: SRR12763776, SRR12763787, SRR12763788), collected in the absence of host contact, were included, averaging ∼14.7 million reads per sample. Reads from both species were merged. Specifically, the first replicate of *Arabidopsis thaliana* was combined with the first replicate of *Cuscuta campestris*, and the same procedure was applied to the other replicates. This resulted in three merged transcriptome datasets: SRR22559142 + SRR12763776, SRR22559143 + SRR12763787, and SRR22559144 + SRR12763788, which were used for downstream analysis. Reference genomes were retrieved from NCBI: *A. thaliana* (RefSeq GCF_000001735.4; ∼119.1 Mb) and *C. campestris* (GenBank GCA_900332095.2; ∼476.8 Mb). The three merged datasets were processed through the inDAGO workflow to generate BAM files. Both input and output sequence files were gzip-compressed, which required greater computational resources compared to handling decompressed files and resulted in significantly longer execution times. Each module’s performance was evaluated using default settings. After the quality control phase, the subsequent filtering module applied a minimum read length threshold of 75 and a quality threshold of 20, with trimming performed on both ends of paired-end reads. Indexing and mapping were performed next using a combined approach. Indexing assumed the generation of a single-segment index, excluding subreads (16 bp k-mers) that appeared more than 100 times from the genome indexing process. Mapping was carried out with default advanced parameters: 14 subreads extracted per read, a consensus threshold of 1, up to 3 allowed mismatches, allowance of 1 multi-mapped read, an indel length of 5, and fragment lengths between 50 and 600 bp.

#### 2.4.2 Dataset 2 - Summarization validation (SEQC human data)

To exclusively assess the performance of the summarization module, BAM or SAM files are required, which can be generated through the alignment module. For this evaluation, real RNA-seq data with known count tables from the SEQC Project (GEO accessions: GSM1156797, GSM1156798, GSM1156799) were used. Specifically, a library from a *Homo sapiens* RNA-seq project (SRA accessions: SRR896663, SRR896664, SRR896665 contain ∼5.7, ∼6.1, and ∼5.6 million paired-end reads, respectively, with an average length of 165 bp.) was downloaded from the Sequence Read Archive (SRA). The dataset consists of paired-end FASTQ files generated on an Illumina HiSeq 2000 platform. The hg38 human genome, along with its corresponding annotation file, provided in FASTA and GTF formats, respectively (RefSeq: GCF_000001405.39; genome size: ∼3.1 Gb) served as the reference for alignment and summarization. Summarization was performed using default parameters, focusing on meta-feature-level assignment and using the “gene_id” attribute in the GTF annotation. This approach minimizes multiple overlaps and excludes multi-mapping reads. The analysis was run in parallel across the three samples, allocating two threads per sample.

#### 2.4.3 Dataset 3 - Exploratory data analysis and DEG identification (mouse mammary gland)

Finally, to evaluate the performance of the data exploration and differential expression analysis modules, read count tables are required, which can be generated through the alignment and the subsequentially summarization module. For this evaluation, we utilized RNA-seq data from luminal and basal mammary epithelium cells collected from the mammary glands of virgin, 18.5-day pregnant, and 2-day lactating mice. This dataset, originally from Fu et al. (2015), is also featured as a case study titled “RNA-Seq profiles of mouse mammary gland” in the edgeR user guide. The sequence and count data are publicly available in the Gene Expression Omnibus (GEO) under accession number GSE60450. After generating read count tables following recommendations from the edgeR user guide, the data were explored through the dedicated inDAGO module. Then differentially expressed genes were identified using default advanced parameters. These included a filtering method based on Chen et al. [20], quasi-likelihood F-tests (QLFTest) for statistical evaluation, and TMM normalization. P-values were adjusted using the false discovery rate (FDR), with significance thresholds set at 0.05 and an absolute log_2_ fold-change (|log2FC|) cutoff of 1.2 to classify genes as up or downregulated. The dataset comprises luminal and basal mammary epithelial cells from adult female mice at three physiological states: virgin, pregnant (E18.5), and lactating (postpartum day 2). Six groups were analyzed: Basal virgin (B_virgin), Basal pregnant (B_pregnant), Basal lactating (B_lactate), Luminal virgin (L_virgin), Luminal pregnant (L_pregnant), and Luminal lactating (L_lactate), each with two biological replicates (total n = 12). The analysis included in pairwise comparisons of B_pregnant vs. B_lactate, B_virgin vs. L_virgin, and B_pregnant vs. L_pregnant.

## 3 Result

### 3.1 Dataset 1 - Dual RNA-seq mapping result (*Arabidopsis* - *Cuscuta*)

**FIGURE 4:**
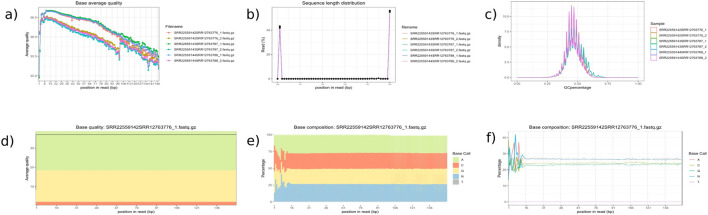
Quality Control Module Outputs. This figure presents key quality control plots generated by inDAGO: **(a)** average base quality line plot; **(b)** sequence length distribution; **(c)** GC content distribution across reads; **(d)** base quality boxplot showing average and variation per base position; **(e)** base composition line plot; and **(f)** base composition area chart across the dataset. Together, these visualizations offer a comprehensive assessment of the quality and characteristics of the raw sequencing data. The analyzed samples include *Arabidopsis thaliana* stem tissue (accessions SRR22559142, SRR22559143, and SRR22559144) and *Cuscuta campestris* tissue (accessions SRR12763776, SRR12763787, and SRR12763788). Reads from each species were paired to create three combined transcriptome datasets: SRR22559142 + SRR12763776 (resulting in SRR22559142SRR12763776_1. fastq.gz and SRR22559142SRR12763776_2. fastq.gz), SRR22559143 + SRR12763787 (SRR22559143SRR12763787_1. fastq.gz and SRR22559143SRR12763787_2. fastq.gz), and SRR22559144 + SRR12763788 (SRR22559144SRR12763788_1. fastq.gz and SRR22559144SRR12763788_2. fastq.gz). Quality assessments for the paired-end reads are presented in the figure panel. Although all samples are displayed in panels **(a–c)**, only the sample SRR22559142SRR12763776_1. fastq.gz is shown in panels **(d–f)**, as the plots are limited to displaying one sample at a time.

We tested the combined mapping workflow on three merged samples created by pairing *A. thaliana* and *C. campestris* replicates. Quality control indicated a mean base quality above Q35, corresponding to an error rate of 0.035%, with an average read length of approximately 150 bp. Base composition along the reads was generally uniform, except for the initial bases affected by hexamer priming during library preparation. Each merged sample contained an average of 36.7 million reads. After filtering, only 0.05% of reads were discarded, and mapping achieved alignment rates above 90% to the reference genomes. Two-sided cross-mapped reads—aligning to both genomes—accounted for less than 0.01%, while reads incorrectly assigned to the wrong genome represented approximately 0.02% of the input (Tables 1, 2). These results are consistent with those reported by Fruggiero et al. (2024), with minor variations attributable to differences in the underlying algorithms. Quality control plots are shown in Figure 4, and filtering summaries are presented in Supplementary Table S1.

**TABLE 1:** Read mapping counts obtained with the combined approach.

Libraries (*Arabidopsis* + *Cuscuta*)	Replicate	Raw reads	Processed reads	Uniquely mapped onto *Arabidopsis*	Uniquely mapped onto *Cuscuta*	Two-side cross-mapped	Unmapped and multiple mapped^a^
SRR22559142 + SRR12763776	Replicate 1	35702825	35685953	19595767 (54.91%)	14811039 (41.5%)	938 (0.003%)	1278209 (3.58%)
SRR22559143 + SRR12763787	Replicate 2	36282091	36261719	20066086 (55.34%)	14801634 (40.82%)	8799 (0.024%)	1385200 (3.82%)
SRR22559144 + SRR12763788	Replicate 3	38034066	38014805	21403053 (56.3%)	15194496 (39.97%)	1,474 (0.004%)	1415782 (3.72%)

a Multi-mapped reads are sequences that align to multiple locations within the reference genome.

**TABLE 2:** Count of reads correctly assigned to their respective genomes.

References genome	Replicate	Unambiguously mapped reads	*Arabidopsis* mapped reads	*Cuscuta* mapped reads
*A. thaliana*	Replicate 1	19595767	19593126 (99.99%)	2,641 (0.01%)
*A. thaliana*	Replicate 2	20066086	20061888 (99.98%)	4198 (0.02%)
*A. thaliana*	Replicate 3	21403053	21399033 (99.98%)	4020 (0.02%)
*C. campestris*	Replicate 1	14811039	714 (0.005%)	14810325 (99.995%)
*C. campestris*	Replicate 2	14801634	10928 (0.07%)	14790706 (99.93%)
*C. campestris*	Replicate 3	15194496	1,472 (0.01%)	15193024 (99.99%)

### 3.2 Dataset 2 - Summarization result (SEQC human data)

To independently evaluate the summarization module, we processed three human RNA-seq samples from the SEQC project. Each sample had an average of 5.8 million aligned reads, with feature assignment successfully capturing approximately 71% of mapped reads under the default counting settings. Comparison of inDAGO’s gene counts to the published SEQC reference showed a mean relative difference of 0.24 across shared genes, indicating strong concordance with established summarization outputs. Per-sample assignment count tables are provided in Supplementary Table S2.

### 3.3 Dataset 3 - Exploratory data analysis and DEG identification (mouse mammary gland)

**FIGURE 5:**
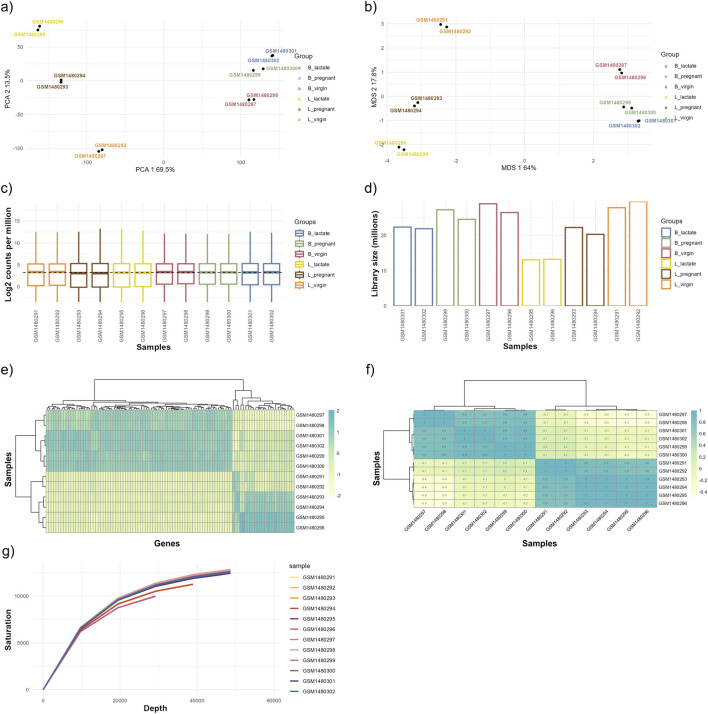
Outputs from the exploratory data analysis (EDA) module. This figure displays graphical outputs produced by the EDA module based on the normalized data, including **(a)** Principal Component Analysis (PCA) plot, **(b)** Multi-Dimensional Scaling (MDS) plot, **(c)** gene expression boxplot, **(d)** library size bar plot, **(e)** gene expression heatmap, **(f)** correlation heatmap, and **(g)** saturation plot. These visualizations enable detailed examination of data distribution, relationships, and overall expression patterns, helping to identify trends, biases, or outliers within the dataset. The GEO accession numbers for each sample group are: L_virgin (GSM1480291, GSM1480292), L_pregnant (GSM1480293, GSM1480294), L_lactate (GSM1480295, GSM1480296), B_virgin (GSM1480297, GSM1480298), B_pregnant (GSM1480299, GSM1480300), and B_lactate (GSM1480301, GSM1480302).

**FIGURE 6:**
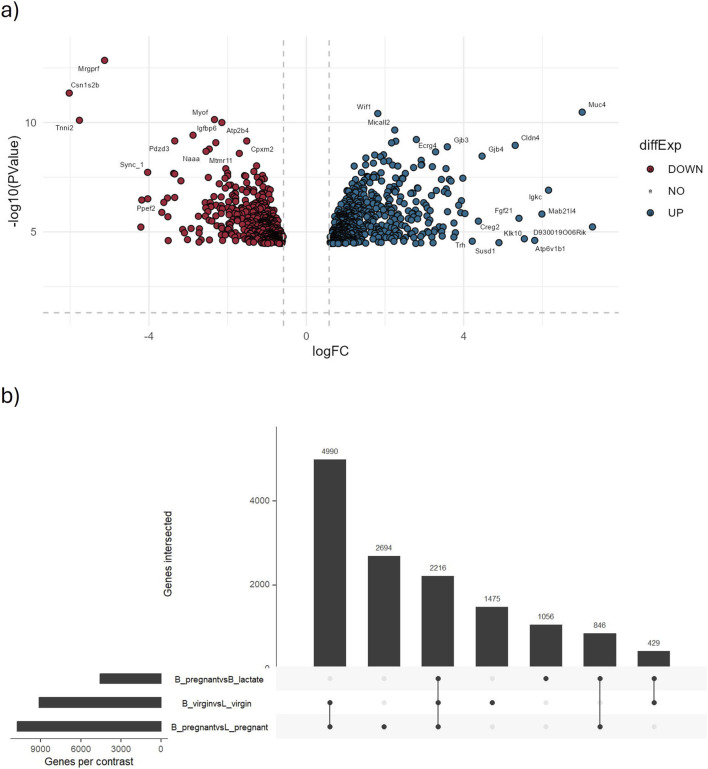
Results from the identification of differentially expressed genes module. This figure displays key graphical outputs generated during the identification of DEGs. **(a)** The volcano plot highlights the top 1,000 most significantly differentially expressed genes from the B_pregnant vs. B_lactate comparison. Each point represents a gene, with the x-axis showing the log2 fold change and the y-axis the negative log10 p-value. The dashed lines indicate the significance thresholds (p-value = 0.05 and |log2 fold change| = 0.58). Genes meeting these criteria are clearly marked, facilitating quick interpretation of up- and downregulated genes. **(b)** The UpSet plot summarizes all considered comparisons, with horizontal bars indicating the number of genes in each set and vertical bars showing the intersections, revealing shared DEGs across comparisons.

We analyzed a publicly available mammary gland RNA-seq dataset using the EDA and differential expression modules to evaluate biological signal detection and statistical performance (Figures 5,6). The dataset includes 12 samples across six groups (B_lactate, B_pregnant, B_virgin, L_lactate, L_pregnant, L_virgin) with two replicates per group.

Dimensionality-reduction analyses (PCA and MDS) clearly separate samples by physiological state and cell type: PC1 explains ∼69.5% of the variance, while PC2 accounts for ∼13.5%, and the MDS axes show a comparable pattern (Figures 5a,b). Separation is more pronounced for luminal (L) samples than for basal (B) samples, indicating stronger condition-specific transcriptional shifts within the luminal population.

Per-sample sequencing depth ranges from ∼12 million to ∼29 million reads: L_lactate samples have the lowest depth (∼12–13M), L_virgin samples the highest (∼27–29M), and basal groups fall in an intermediate range (∼21–27M). Replicates within each condition are closely matched (differences of only ∼1–2M reads), indicating good technical reproducibility, although between-condition differences in library size could affect sensitivity for low-abundance transcripts (Figure 5d). Boxplots of log2 counts per million (CPM) show broadly comparable distributions across all samples (Figure 5c).

Sample clustering in the expression and sample–sample correlation heatmaps confirms that luminal and basal samples form distinct clusters and that biological replicates are consistent (within-group correlations are high relative to between-group correlations; Figures 5e,f). The sequencing saturation curve indicates that a clear plateau has not been fully reached, suggesting diminishing but non-negligible returns from additional sequencing depth and that further sequencing could recover additional low-abundance genes (Figure 5g).

Out of 42,396 genes tested, ∼4,700 were identified as differentially expressed (DEGs) using |log2FC| > 1.2 and FDR <0.05. In the B_pregnant vs. B_lactate contrast (volcano plot, Figure 6a), genes with both large fold changes and strong statistical significance are highlighted. Among these, 46 genes exhibit very strong upregulation (log2FC > 4 and FDR <0.05), while 16 show very strong downregulation (log2FC < −4 and FDR <0.05).

The UpSet plot summarizes DEG overlap across comparisons (Figure 6b): 2,216 DEGs are shared among the three comparisons considered, with the B_pregnant vs. L_pregnant contrast contributing the largest set of uniquely regulated genes (2,694 unique DEGs). These results are consistent with outcomes obtained following the edgeR User’s Guide (https://bioconductor.org/packages/release/bioc/vignettes/edgeR/inst/doc/edgeRUsersGuide.pdf). Complete DEG statistics are provided in Supplementary Table S3.

### 3.4 Computational performance

Execution times for each module are summarized in Table 3. All runs were performed using default parameters unless otherwise noted. For the mapping benchmark, compressed input and output files were used, which is expected to increase processing time compared to workflows with uncompressed files.

**TABLE 3:** Stepwise execution times (minutes) recorded on three laptops running different operating systems.

Modules		Parallel sampling^a^	Operating systems		
			GNU-linux^b^	Windows^c^	macOS^d^
Dataset 1	Quality control	1	7,5	7,6	4,0
	Filtering	3	43,1	47.53	34.3
	Indexing	1	8,75	14,1	4,7
	Mapping	1	384,20	462,42	354,1
Dataset 2	Summarization	3	0,7	1,3	0,4
Dataset 3	Exploratory data analysis	1	<0,1	<0,1	<0,1
	Differential expression analysis	1	0,2	0,2	0,1

Execution times correspond to the specific datasets used. For details on data sizes, please refer to the Materials and Methods section.

a This refers to conducting the analysis on one or multiple samples simultaneously.

b GNU/Linux distribution Ubuntu 20.04.6 LTS (Focal Fossa) with an Intel® Core™ i7-1185G7 processor and 16 GB, of RAM (1,600 MHz).

c Windows 11 with an Intel® Core™ i5-9300HF, processor and 16 GB, of RAM (2,400 MHz).

d macOS, Sequoia version 15.5 with an Apple M1 chip and 16 GB, of RAM (2,666 MHz).

## 4 Discussion

This work introduces inDAGO, a standalone, cross-platform software tool with an intuitive graphical user interface that eliminates the need for programming expertise. By lowering the technical barrier, inDAGO exemplifies the democratization of bioinformatics, addressing the critical need to make advanced computational tools accessible to researchers regardless of their coding background (Krampis, 2022). Many biologists face significant challenges navigating complex data analysis workflows, making user-friendly interfaces essential to overcoming these obstacles. Open-source platforms that integrate diverse bioinformatics tools into streamlined workflows simplify the analysis of high-throughput sequencing data. By emphasizing ease of use and accessibility, such platforms empower a broader community of researchers to independently perform sophisticated analyses, fostering inclusivity and accelerating scientific progress.

inDAGO supports two types of transcriptomic analyses: dual RNA-seq, which can be performed in either sequential or combined modes, and bulk RNA-seq, both starting directly from raw sequencing data. To evaluate its performance and reliability, we tested inDAGO on public datasets across three operating systems, using a standard laptop with 16 GB of RAM. The results demonstrated that all modules functioned efficiently and as expected. In particular, the read discrimination results were consistent with those reported by Fruggiero et al. (2024), with minor differences attributable to variations in the underlying algorithms. Specifically, Trimmomatic (Bolger et al., 2014) and STAR (Dobin et al., 2013) were used instead of the embedded tools in inDAGO (primarily: ShortRead, Biostrings, and Rsubread). The summarization achieved an assignment rate comparable to what can be obtained by following the Rsubread user’s guide pipeline. The minor discrepancies between the results and the SEQC project likely stem from slight algorithmic and parameter differences. While the results of DEG analysis are consistent with those reported in the edgeR manual. Indeed, the MDS and PCA plots show replicates from the same experimental group clustering together, reflecting their biological similarity, while samples from different groups form separate clusters, indicating significant condition-specific differences. This pattern suggests that inter-group variability exceeds intra-group variation, highlighting meaningful differential expressions. These results demonstrate that inDAGO produces outputs suitable for downstream biological interpretation and can serve as a reliable end-to-end solution for typical dual and bulk RNA-seq experiments. Benchmarking against standard command-line workflows shows largely equivalent results, with minor differences due to default parameter choices affecting a small fraction of ambiguous reads. For routine analyses, users can expect concordant biological conclusions regardless of workflow.

For cases requiring strict minimization of cross-mapping, such as interacting organisms with highly similar genomes, more conservative settings (e.g., stricter mapping parameters in a combined approach) are recommended, improving accuracy (Fruggiero et al., 2024).

While inDAGO does not yet include integrated gene-set enrichment, co-expression modules, or dedicated single-cell/non-coding RNA workflows, it exports standardized count and DEG tables compatible with popular downstream tools. Future development will prioritize these additions alongside expanded statistical options for DEG analysis.

Computational constraints are mitigated via chunked and parallelized processing of large genomes and deep sequencing datasets, enabling analysis on typical laptops. Therefore, processing datasets larger than those used in the evaluation section results in a linear increase in execution time, without risking memory overflow. For projects aiming for maximum sensitivity in gene discovery, users can export inDAGO’s intermediate files and re-run alignment or quantification with alternative parameters. The software is intentionally designed to facilitate these handoffs. By providing comprehensive intermediate, BAM, and count files, inDAGO enables transparent reporting while allowing users to combine GUI-driven convenience with command-line precision. Practical users will also benefit from the detailed guidance provided in the documentation. In summary, inDAGO is a robust and powerful tool that guides scientists through the complexities of dual and bulk RNA-seq data analysis. Upcoming versions of inDAGO will add new modules and enhanced statistical approaches for downstream analyses.

## Data Availability

The original contributions presented in the study are included in the article/Supplementary Material, further inquiries can be directed to the corresponding author.
